# Case Report: Clinical Treatment of the First Critical Patient With Coronavirus Disease (COVID-19) in Liaocheng, Shandong Province

**DOI:** 10.3389/fmed.2020.00249

**Published:** 2020-05-28

**Authors:** Hui Tian, Yuanda Sui, Suochen Tian, Xiuli Zou, Zhiping Xu, Huang He, Tiejun Wu

**Affiliations:** Department of Critical Care Medicine, Liaocheng People's Hospital, Liaocheng, China

**Keywords:** COVID-19, critical COVID-19 patient, clinical characteristics, clinical diagnosis and treatment, plasma exchange, high-flow nasal cannula, case report

## Abstract

This paper reports the clinical characteristics, diagnosis, and treatment of the first critical COVID-19 patient in Liaocheng City, who was admitted to the intensive care unit isolation ward of Liaocheng People's Hospital on February 11, 2020. On admission, the patient had difficulty breathing, the oxygenation index was 135 mmHg, and the blood lactate was 5.6 mmol/L. After comprehensive treatment including high-flow nasal cannula oxygen therapy, plasma exchange, antiviral and anti-infection therapies, immune regulation, liquid volume management, glucocorticoid, enteral nutrition support, analgesia and sedation, blood glucose control, anticoagulation and thrombus prevention, and electrolyte balance maintenance, the patient was finally cured, and discharged. The purpose of this case report is to provide a reference for the clinical diagnosis and treatment of critical COVID-19 patients.

## Introduction

Since December 2019 cases of the novel coronavirus disease (COVID-19) (1) were reported in Wuhan, Hubei Province, and the disease soon spread to the rest of China. The initial symptoms were mostly fever, weakness, and dry cough, while symptoms such as dyspnea gradually appeared. In critical cases, acute respiratory distress syndrome or septic shock and even death could occur (1–3). Current therapeutic strategies focus on isolation and organ support therapy. On February 29, 2020, 756 cases had been reported in the Shandong Province (including 7 severely ill cases, 4 critically ill cases, and 6 deaths), including 38 in Liaocheng City. Among these, a critical patient was admitted to Liaocheng People's hospital on February 11, 2020, and this report describes the clinical characteristics, treatment, and outcome of this patient.

## Case Presentation

A male, 54-year-old patient with body mass index 25.7 kg/m^2^ was admitted to the intensive care unit (ICU) isolation ward of Liaocheng People's Hospital after 8 days of fever and 7 days of coughing.

No accurate contact history was available. The patient had been diagnosed with diabetes 2 years earlier and had been on oral metformin (DMBG). No details were available about blood glucose control.

The patient developed a fever with no apparent triggers on February 3, 2020, with a highest recorded body temperature of 38.0°C. He had no chills or shivering, and developed a cough on February 4, with yellow-colored sputum accompanied by mild chest tightness and pain, fatigue, and discomfort. The symptoms were not relieved by traditional Chinese medicine, and he was admitted to the local hospital on February 7. CT scan on admission showed inflammatory affections on both lungs. The patient was given anti-inflammatory and anti-viral treatments. On February 9 he tested positive to the pharyngeal swab COVID-19 nucleic acid test and was transferred to the airborne-isolation ward of Liaocheng Infectious Disease Hospital for further treatment and quarantine. On February 10 his highest temperature was 39.0°C and cough with sputum and chest tightness persisted; transcutaneous oxygen saturation was 93% (oxygen uptake of 2 L/min). On February 11 breathing became more difficult and chest tightness worsened. Arterial blood gas analysis (oxygen uptake of 4 L/min) reported the following: pH, 7.46; PaCO_2_, 26 mmHg; PaO_2_, 50 mmHg; blood lactate (Lac), 5.6 mmol/L; and oxygenation index (OI), 135 mmHg. The patient was then transferred to the ICU isolation ward of Liaocheng People's Hospital at 23:45 on February 11.

On February 12 (Day 1 of hospitalization to ICU isolation ward of Liaocheng People's Hospital) body temperature was 36.9°C, heart rate 81 bpm, respiratory frequency 35/min, and blood pressure 141/87 mmHg. The patient was conscious but nervous, and showed hyperventilation and lip cyanosis. The breathing sound was thick on both lungs, without obvious dry or wet rales. The heart rate was regular, the abdomen was flat and soft, without tender or rebound pain. There was no edema on either leg, and hands and feet were warm.

### Supplementary examinations

On February 9, a pharyngeal swab COVID-19 nucleic acid test performed at the Liaocheng Center for Disease Control (CDC) was positive.

On February 12 blood test results were as follows: white blood cells (WBC), 7.62 × 10^9^/L; neutrophils (NE), 6.98 × 10^9^/L; neutrophil percentage (NEU%), 91.7%; lymphocytes (LYM), 0.30 × 10^9^/L; platelets (PLT), 282 × 10^9^/L; C-reactive protein (CRP), 88.0 mg/L; erythrocyte sedimentation rate (ESR), 80 mm/h; procalcitonin (PCT), 0.78 ng/mL; D-dimer, 0.72 ug/mL; CD3+ T cells, 175 × 10^3^/ml; CD4+ T cells: 79 × 10^3^/ml; CD8+ T cells, 95 × 10^3^/ml; CD4/CD8 ratio, 0.83; albumin, 31g/L; creatinine, 52 μmol/L. Troponin I (cTn I), brain natriuretic peptide (BNP), creatine kinase (CK) and blood urea nitrogen (BUN) were normal.

Arterial blood gas analysis gave the following values: pH, 7.43; PCO_2_, 32.9 mmHg; PO_2_, 84 mmHg, Na^+^, 144 mmol/L; K^+^, 3.56 mmol/L; Hb, 10.4 g/dL; Lac, 2.8 mmol/L; ${\text{HCO}}_{3}^{-}$, 22.9 mmol/L (with high-flow nasal cannula (HFNC) for 2 h, flow velocity of 45 L/min, and FiO_2_ 60%); OI, 140 mmHg.

**Figure 1:**
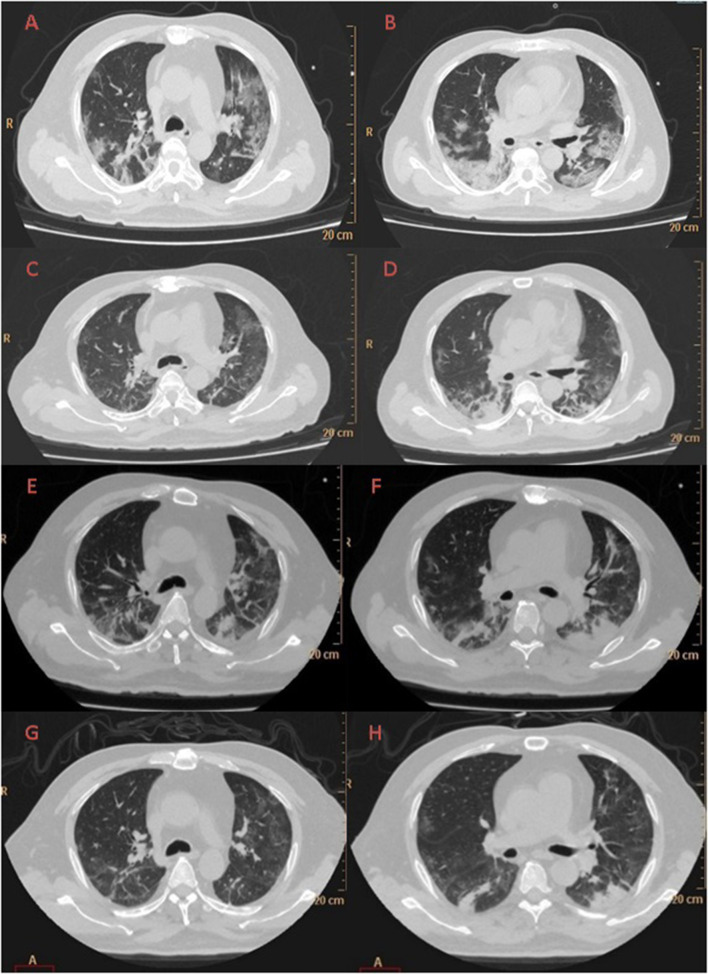
Evolution of the chest CT of the COVID-19 patient during hospitalization to ICU isolation ward of Liaocheng People-s Hospital: Day 1 **(A,B)** A large area of ground-glass opacity with uneven density was seen in the subpleural region of both lungs, with fine grid (crazy-paving sign), predominantly in the lower lobes. Multiple patchy consolidations were apparent in the lingular segment of the left upper lobe and bilateral lower lobes, with air bronchus-charging sign and thickening of the pulmonary interstitium surrounding the lesions. Day 6 **(C,D)** Patchy ground-glass opacity was seen in the subpleural region of both lungs, with multiple chords and consolidation shades in the bilateral lower lobes, but the extent decreased and the density thinned. Day 12 **(E,F)** There were reduced regions of initial ground-glass opacity, with new area of subpleural consolidation. Day 19 **(G,H)** Most of ground-glass opacity lightened or disappeared, partial area of consolidation was still observed.

A large area of ground-glass opacity with uneven density was seen on chest CT on February 12 in the subpleural region of both lungs, with fine grid (crazy-paving sign), predominantly in the lower lobes. Multiple patchy consolidations were apparent in the lingular segment of the left upper lobe and bilateral lower lobes, with air bronchus-charging sign and thickening of the pulmonary interstitium surrounding the lesions (Figures 1A,B).

## Interventions

### Mechanical Ventilation and Oxygen Therapy

The patient was treated with HFNC with flow velocity of 45 L/min, and the respiratory distress stopped worsening, with FiO_2_ falling from 60 to 50% on day 2, and to 40% on day 4. On day 5 OI increased to 328 mmHg. On day 6 oxygen inhalation through nasal catheter was used with a velocity of 3 L/min, and OI was 288 mmHg. After 36 h the heart rate increased, the cough became heavier, and OI fell to 209 mmHg. HFNC was then reapplied with a velocity of 40 L/min and FiO_2_ 35%. On day 15 the velocity of oxygen inhalation through nasal catheter was 3L/min, falling to 1 L/min on day 17, until oxygen inhalation was terminated on day 19.

### Plasma Exchange

The patient was treated with plasma exchange, 12 h after hospitalization, by Fresenius (Germany) multifiltrate bedside blood purifier and Fresenius P2 plasma separator, processing 2000 ml of blood plasma in 120 min. The process was smooth and the patient did not have fever, shivers from cold, or rashes.

### Anti-viral Therapy

On February 12 Ribavirin (RBV) 500 mg was administered by intravenous drip infusion 2 times per day for 4 days; on February 12 umifenovir 0.2 g was administered orally 3 times per day for 2 days, and recombinant human interferon α-2b (5 million units) by aerosol inhalation 2 times per day for 7 days.

### Anti-infection Therapy

On admission (on February 12) the patient was given imipenem–cilastatin (1.0 g) by intravenous drip infusion once every 8 h for 3 days; on day 4 ceftriaxone sodium (2.0 g) was given instead by intravenous drip infusion once per day for 4 days. On day 8 the antibiotics was changed to cefoperazone–sulbactam (3.0 g) by intravenous drip infusion once every 8 h for 7 days. On day 9 linezolid (600 mg) was added by intravenous drip infusion once every 12 h for 6 days, and on day 9 the first dose of caspofungin by intravenous drip infusion was 70 mg, and later 50 mg were given once per day for 10 days. On day 15 cefoperazone–sulbactam and linezolid were discontinued and levofloxacin (500 mg) was administered by intravenous drip infusion once per day for 5 days. On day 19 caspofungin was discontinued.

### Immunomodulating Therapy

Starting on day 1 thymalfasin 1.6 mg was subcutaneously injected every 12 h for 14 days, and once per day for 5 days starting on day 15. Starting on day 1 immune globulin 10.0 g was administered by intravenous drip infusion once per day for 10 days.

### Glucocorticoid Therapy

On day 1 methylprednisolone 40 mg was administered by intravenous drip infusion every 8 h. On day 2 it was reduced to every 12 h for 3 days, and once a day from day 5 for 2 days, until it was discontinued on day 7.

### Anticoagulation Therapy

On day 2 D-dimer coagulation increased. The patient was lying in bed and catheterization of the femoral vein was applied. To avoid the formation of deep venous thrombosis, enoxaparin 5000U was added by subcutaneous injection every 12 h for 16 days. The D-dimer was high throughout the course, the highest value being 3.26 ug/ml.

### Liquid Volume Management

Liquid volume was monitored by bedside ultrasound to prevent the increase of lung water. From day 1 furosemide 10 mg was intravenously injected every 12 h for 2 days; on day 3 it was changed to spironolactone 20 mg and hydrochlorothiazide 25 mg, both administered orally twice per day.

### Nutritional Support

The patient had a history of diabetes and developed serious gastro–intestinal symptoms after admission. From day 2 dieticians performed nutritional risk screening and dietary intake assessments, and the nutritional therapy plan was made according to guidelines (4) and clinical experience. The daily energy input was 20–25 Kcal/kg, and that of protein 1–1.5 g/kg. Nutritional support therapy not only meets the energy and protein requirements, but also guarantees blood glucose stability, liquid balance, and gastrointestinal tolerance.

### Other

Sedation, analgesia, humanistic care, early-stage physical therapy, traditional Chinese medicine therapy, and blood glucose control were administered as well.

### Outcomes

**Figure 2:**
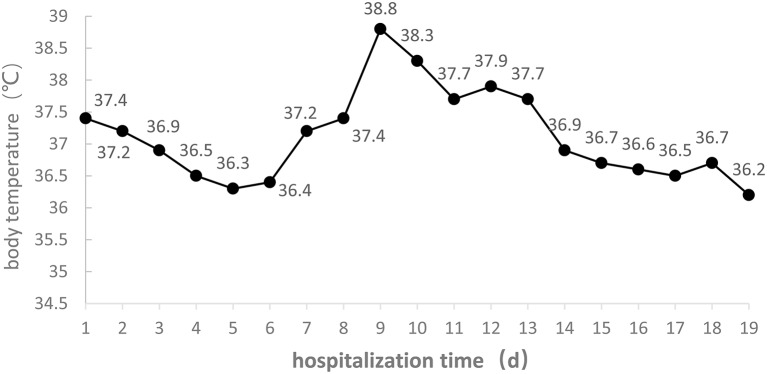
Body temperature of the COVID-19 patient during the 19 days of hospitalization to ICU isolation ward of Liaocheng People's Hospital.

On admission the patient had dry cough without sputum; when moving or changing body position the cough became heavier, but breathing did not become more difficult, and chest tightness was not more severe. On day 7, after oxygen inhalation through nasal catheter, the cough worsened. On day 9 there was yellow-colored sputum with blood, which later increased. On day 14 there was an obvious decrease of sputum. The daily changes of body temperature are shown in Figure 2.

**Figure 3:**
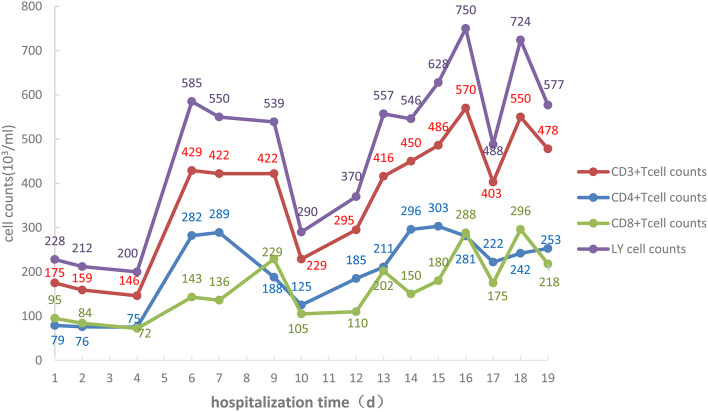
Absolute values of lymphocytes, CD3+ T cells, CD4+ T cells, and CD8+ T cells of the COVID-19 patient during the 19 days of hospitalization to ICU isolation ward of Liaocheng People's Hospital.

**Figure 4:**
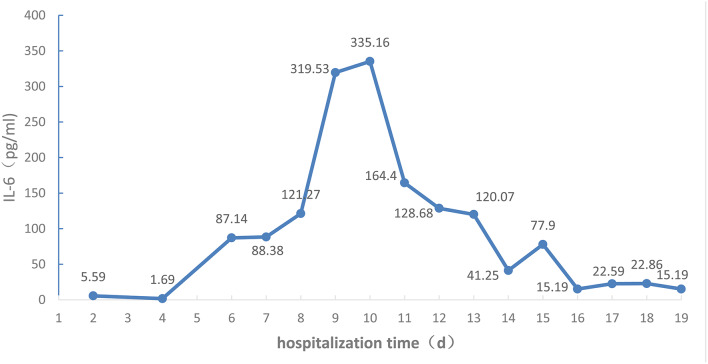
IL-6 levels of the COVID-19 patient during the 19 days of hospitalization to ICU isolation ward of Liaocheng People's Hospital.

Changes in absolute values of lymphocytes, CD3+ T cells, CD4+ T cells, and CD8+ T cells are shown in Figure 3, while those of IL-6 are shown in Figure 4.

Although the pharyngeal COVID-19 swab before hospitalization to Liaocheng Infectious Disease Hospital had been positive, two whole-blood COVID-19 tests on day 2 and 3 after admission to Liaocheng People's Hospital were negative, as were four pharyngeal swab COVID-19 nucleic acid tests from day 3 to 6 and the anal swab COVID-19 test on day 5. Sputum culture revealed a normal flora, and blood culture showed no bacterial growth. On day 18 the results of the COVID-19 serum antibodies IgG and IgM qualitative analyses were both positive.

Patchy ground-glass opacity was seen on CT in the subpleural region of both lungs on day 6, with multiple chords and consolidation shades in the bilateral lower lobes, but the extent decreased and the density thinned (Figures 1C,D). The same was observed, with increased extent, on day 12 (Figures 1E,F). On day 19 the lungs appeared much improved on CT (see Figures 1G,H).

After hospitalization blood pressure was stable, heartbeat and blood lactate were normal, and there was no circulatory dysfunction. Hemobilirubin was slightly elevated, aminotransferases were normal, creatinine was low, and urea nitrogen initially increased but gradually became normal. After enoxaparin was applied D-dimer remained elevated. Thromboelastograms (TEG) on day 3 and 17 showed that the clotting status was normal.

### Clinical Outcome

On the afternoon of day 1 a remote consultation conference was held with the COVID-19 expert team of the Shandong Province. The severity evaluation was adjusted from critical illness to severe illness, and to moderate illness on day 6. On day 20 the patient was cured and discharged. At the time of discharge, the patient was in good spirits, with stable breathing, no cough or expectoration, good nutrition and sleep, and normal fasting and postprandial blood sugar. During 30 days of follow-up, blood analysis showed all indexes returned to normal, significantly improved chest CT, and no complications.

Informed consent to publication was obtained from the patient.

## Discussion

This is a confirmed COVID-19 case. On admission the patient had difficulty breathing, with OI <150 mmHg, peripheral blood lymphocyte count decreased to 0.30 × 10^9^/L, blood lactate as high as 5.6 mmol/L, and extensive lesion range on CT, indicating that the health of the patient was worsening quickly and the severity was critical (2, 5). The patient was given a comprehensive therapy of HFNC, plasma exchange, bedside ultrasound volume management, early-stage intestinal nutrition, immunomodulating therapy, sedation, analgesia, and physical therapy. Invasive mechanic ventilation was avoided, and the patient was cured and discharged. The experience gained from the treatment of the patient can be summarized as follows.

It is important to assess the severity grade as early as possible and take measures to prevent its worsening. Attention should be paid to changes in clinical warning indexes (5): The Acute Physiology And Chronic Health Evaluation II (APACHE II) (6) and the pneumonia severity index (PSI) grading systems (7) should be applied to evaluate the severity of the illness (8, 9). On admission, the APACHE II index was 21, and it decreased to 16 on day 2, indicating improved conditions. On admission, the PSI index was 134, indicating high risk, but decreased to 84, indicating low risk, on day 2. These two grading indexes are concordant, and can be used in severity evaluation, risk assessment, and the early identification of patients with severe and critical illness.

Ventilation therapy should be given to the early-identified severe and critical cases to avoid worsening organ damage caused by anoxia. On admission, the patient had respiratory distress and OI <150 mmHg, so he was given HFNC immediately. Recently HFNC has been widely used in clinical treatment and its clinical effectiveness in the treatment of mild to moderate respiratory failure has been established (10). HFNC played a key role in the correction of the patient's early respiratory failure, despite of the fact that the OI was lower than 150 mmHg on days 1 and 2, as the patient showed less nervousness and anxiety, breathing tightness was lessened, vital signs were stable, and blood lactate was normal. The OI increased gradually to more than 300 mmHg on day 5, and on day 6 HFNC was discontinued. After being given oxygen inhalation through nasal catheter, the patient had more difficulty breathing and coughed more heavily, and the heart rate increased. After HFNC-assisted respiration with low parameter the patient's discomfort was soon relieved. Therefore, HFNC played an important role in improving and maintaining the respiratory functions of this patient in the later phase.

Blood plasma exchanges eliminated the inflammatory factors and blocked the “cytokine storm” to relieve the damage to the organism caused by inflammatory reactions, restraining the development of the disease. Studies have demonstrated serious inflammatory reactions inside the bodies of COVID-19 patients, especially those in severe and critical conditions (3, 11), and that the cytokine storm correlates with disease severity (1). Available guidelines (5, 12) suggest that extracorporeal blood purification, including plasma exchanges, adsorption, perfusion, and blood/plasma filtration, should be used in critically ill patients with severe inflammatory reactions. At the early stage the severity level changed quickly from moderate to critical, presumably due to the cytokine storm. After admission the patient was given timely plasma exchange therapy, after which only the IL-6 index was slightly elevated, the other inflammatory factors such as IL-2, IL-4, IL-10, IL-17A, and TNF-a being all in the normal range. As no monitoring of inflammatory factors had been performed before treatment, no comparison is possible. However, dynamic monitoring found that IL-6 increased with time, indicating persistent inflammation after plasma exchange, and, indirectly, that plasma exchanges can eliminate the inflammatory medium and restrain inflammatory reactions. After the treatment the APACHE II and PSI indexes were lowered, and the patient overall condition was improved.

Attention should be paid to immunomodulating therapy, and inflammatory factors and immune cells should be monitored during anti-viral and antibiotic treatment to provide a basis for anti-inflammatory treatment, as the immune system is attacked by COVID-19. On admission, the lymphocyte count was 0.30 × 10^9^/L, and the absolute values of all T cell subsets were obviously decreased. CD4/CD8 reversal indicated restraints to immunity, and the patient was given thymalfasin and immune globulin to regulate the immune functions. On February 10 the body temperature was 39°C and on February 12 blood analysis showed a neutrophil percentage of 91.7%, so that imipenem–ilastatin was administered as anti-bacterial drug. On day 4 the anti-bacterial drug was downgraded to ceftriaxone sodium and the body temperature was normal during the administration of this drug, while all lymphocyte counts increased. On days 9 and 10 the fever reappeared and the patient coughed yellow-colored sputum. IL-6 increased and all lymphocyte counts decreased. On day 12 the chest CT scan showed the expansion of ground-glass opacities, solidification, and stripes, considered to result from a new infection, which was a key cause of the worsening of inflammation and the decrease of immune functions. The lungs were the infected site, but external bloodstream infection was not excluded. The pathogenic agents might have been cocci and fungi. The anti-infection plan was thus changed, and cefoperazone–sulbactam, linezolid, and caspofungin were used to fully cover gram-negative bacilli, positive cocci, and fungi, while thymalfasin was still used to regulate immunity. On day 14 the body temperature was normal and the fever never reappeared. The lymphocyte count and all subset T cell counts gradually increased, while IL-6 decreased, indicating reduced inflammatory reactions and stabilized immune situation after the infections were brought under control. On day 19 the chest CT scan showed obvious improvement.

Attention should also be paid to liquid volume management, to maintain the electrolyte and acid–base balances and allow a stable internal environment. Timely, efficient, and safe supportive therapy is crucial for the treatment of severe cases of COVID-19 (13), in agreement with the accepted philosophy of critical care medicine. Attention should be paid to the management of bedside ultrasound volume to maintain the negative balance and the intake–output balance, and avoid an excessively positive liquid balance which could prevent oxygenation through aggravated pulmonary inflammation.

Notwithstanding the retrospective nature of this case report and the short follow-up, we obtained a valuable patient perspective. The patient felt well and did not have complications. In addition, we believe the most valuable insight derived from this case is the idea of “prevention beforehand”: We applied the APACHEII and PSI grading systems and a series of monitoring indexes to identify severe illness in the early stage, and, especially, we adopted a comprehensive treatment strategy. These approaches blocked the development of the disease, and allowed us to save this critical COVID-19 patient.

However, we have only treated this one patient, whose evolution confirms the effectiveness of the treatment strategy adopted. In order to provide guidance for such cases in the future, we believe our observations could be made robust by appropriate randomized controlled trials.

## Data Availability Statement

The datasets presented in this study can be found in online repositories. The names of the repository/repositories and accession number(s) can be found in the article/Supplementary Material.

## Ethics Statement

The studies involving human participants were reviewed and approved by Liaocheng People's Hospital Ethical Review of Medical Research on Human Being. Written informed consent for participation was not required for this study in accordance with the national legislation and the institutional requirements. Written informed consent was obtained from the individual(s) for the publication of any potentially identifiable images or data included in this article.

## Author Contributions

TW and HT had full access to all of the data in the study and takes responsibility for the integrity of the data and the accuracy of the data analysis. TW, HT, and YS designed the study and wrote the paper. ST and XZ acquisition, analysis, or interpretation of the data. HH and ZX contributed to revision of the manuscript for important intellectual content.

## Conflict of Interest

The authors declare that the research was conducted in the absence of any commercial or financial relationships that could be construed as a potential conflict of interest.
